# Auguste François Chomel (1788–1858) and his Work on Rheumatism: Introducing Rheumatic Heart

**DOI:** 10.31138/mjr.030223.afc

**Published:** 2024-02-12

**Authors:** Georgia Fragou, George Philippidis, Panagiotis Georgakopoulos, Evaggelos Mavrommatis

**Affiliations:** Medical School, National and Kapodistrian University of Athens, Athens, Greece

**Keywords:** John Abercrombie, endocarditis, pericarditis, elaterium, heart inflammation

## Abstract

In the early 19^th^ century, Auguste François Chomel gathered the knowledge on rheumatism, systematised it, and published it in his majestic work *Pathologie* Générale. In his treatise, for the first time, rheumatic heart was discussed. Taking into account the opinions of the ancient Greek physicians, he had described the disease as an acute or chronic manifestation of the inflammation of the heart due to rheumatism. His publication rendered rheumatic heart an issue in vogue for his era, a disease being mentioned in all textbooks of the internal pathology.

## INTRODUCTION

**Figure 1.**
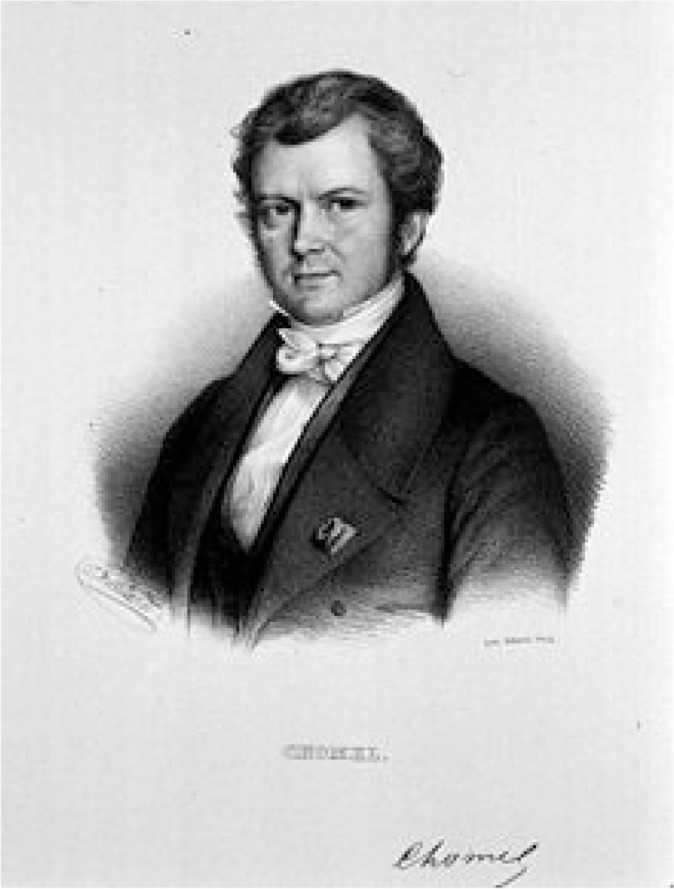
Auguste François Chomel (1788–1858). The National Library of Medicine believes this figure to be in the public domain.

The term rheumatism derives from the ancient Greek word “ρεύμα” (rheuma), meaning something which flows. This term was used by ancient Greek physicians in order to describe a pathological condition in which humor was discharged from one part of the body to the other, provoking mainly swelling or an outflow from either the mouth or the nose, the eyes, the ears, the genitalia, even from the anus.^1^ This concept of humor which flows through the human body endured in medical though until the era of 18^th^–19^th^ centuries.^2^ Anatomical pathology of the medical thought which was developed in Parisian Medical School of the time, had tried to link various conditions provoking swelling or outflow of all type of liquids of the human body with other symptoms. These simultaneously presented symptoms composed a clinical image to be and examined mainly under the new tool of the era, the microscope.^3^ Nevertheless, rheuma having as its main symptom local painful oedemas had remained as the basic clinical manifestation with a traditional perspective linked to the modern concept of rheumatic diseases, mainly for historical reasons. Auguste François Chomel (1788–1858) (**Figure 1**) was among the first to gather all knowledge available on the rheumatism to write a treatise based on its pathology. He had introduced the Rheumatism of the Heart to connect rheumatic diseases with cardiac lesions. For this historical vignette, documentary research was conducted to unveil Chomel’s work.

## LIFE AND WORK

Auguste François Chomel (1788–1858) was an eminent French physician. He was born in Paris during 1788, being a member of a family which had a tradition in producing distinguished physicians and writers. He was the last descendant of Pierre-Jean-Baptiste Chomel (1671–1740), author of *Plantes usuelles* (1712), student and friend of Joseph Pitton de Tournefort (1656–1708), one of the deans of the former Faculty of Medicine in Paris, and the founder of the Parisian School of Pharmacy. His father, Jean-Baptiste Louis Chomel, had been noticed in contemporary literature. Auguste Chomel’s early education had been strong and serious. His family brought him, about the age of twelve, into the Savouré Institution, where he had started his medical studies at the age of eighteen and defended his thesis in February 1813. In 1814, after the wars of the empire, a medical service was entrusted to him in Val-de-Grâce. Entering the hospital career early, he had been firstly attached to Hôpital de la Charité being appointed as an ordinary physician of this establishment. Chomel was granted the position of Professor in 1827. After Laennec’s death, Chomel succeeded him in the chair of medical clinic at the majestic Hôtel-Dieu. Meanwhile, he was a member of the Higher Council of Public Instruction.^4^

In 1817 Chomel published the first edition of his most important and referenced work, his masterpiece *Pathologie G*énérale.^5^ He had endeavoured to define the terms used in medicine in his era and thus to initiate physicians to a more scientific medical language. Towards the beginning of 1820, Chomel submitted to the Society of the Faculty of Medicine of Paris a treatise entitled *Mémoire sur l’existence des Fièvres*.^6^ Some years later, Chomel was elected as an associate member of the Academy of Medicine on 16 April 1823 and a full member on November 5^th^, 1826. He was an important member of the Anatomic pathology movement of the early 19th century France. This movement was based on the scientific research of three magnificent physicians and anatomists, Xavier Bichat (1771–1802), René-Théophile-Hyacinthe Laennec (1781–1826) and Gaspard Laurent Bayle (1774–1816). In the spring of 1828, he had described an epidemic of acute polyneuritis, which is believed to be the first description of the later known as *Guillain-Barré-Strohl* syndrome.^7^ According to the opinion of the American physician Worthington Hooker (1806–1867), Chomel was the first to use for the first time in modern Western medicine the axiom *Primum non nocere* (First, do no harm).^8^ This concept was introduced in medical history by Hippocrates and expressed in Corpus Hippocraticum in the work about *Epidemics* in the form that a physician should obey two principals in his work, to benefit the patient and to not harm him (*Corpus hippocraticum. De morbis popularibus 1.2.5.9–10*).

## RHEUMATISM AND RHEUMATIC HEART

Chomel had understood the complex theory of rheumatism, having described it in detail. He had tried to list the pathological conditions of every part of the human body which could present similar signs as swelling (enema), pain and efflux, especially bleeding, conditions which should be distinguished from the phenomenon of rheumatism. Therefore, he had written on Articular Rheumatism, Visceral Rheumatism, Rheumatism of the Diaphragm, Rheumatism of the Air Tubes, Rheumatism of the Alimentary Canal, Rheumatism of the Bladder, Rheumatism of the Uterus, and Rheumatism of the Heart. His writing was more focused and explanatory on the phenomenon, as he had succeeded to co-examine the anatomical pathology of all these conditions while trying to present a differential diagnosis of entities with similar symptoms. To explore ways of provocation from different aetiology, he had composed a very useful guide for clinical physicians.^9^

Chomel believed that the Rheumatism of the Heart could have been presented as an acute or chronic disease. He had also pointed out that in many cases when diseases of the heart were considered incurable, but were finally cured, these should have been listed probably as rheumatic. He noted that most of the cases concerned heart conditions observed in children or young adults. Therefore, he had suggested much more attention that should be given in those young patients who were born to rheumatic parents, or if they presented rheumatic signs and symptoms for the first time. For the rest of the age groups, he marked that when a cluster of symptoms such as recovered pericarditis, syncope of short duration, or analogous palpitation especially in acute articular rheumatism, or any other heart symptom which disappears after two or three hours, those were cases of probable manifestations of acuter cardiac rheumatism. For the chronic form of rheumatic heart disease, he had mentioned symptoms such as precordial pains, mainly of sudden appearance and mostly during nighttime, independent from activities like walking or climbing stairs and other simultaneous symptoms, such as swellings (oedemas), or disturbed blood circulation. So, he had beautifully distinguished the Rheumatism of the Heart from pleurodynia, pleuritis, nervous palpitation, various organic diseases of the heart, and angina pectoris.^10^

## DISCUSSION

In 1832 Goodrich noted that rheumatism could cause a permanent cardiac impairment, as the weakened heart muscles presented difficulty to pump blood to supply the whole body, and the sufferer feels weakness and tiredness.^11^ Macleod in 1842 had strongly connected rheumatic fever with heart damage.^12^ The same year, 1817, of the *Pathologie* Générale publication, Scottish physician John Abercrombie (1780–1844) published a case series under the title *Contributions on the pathology of the Heart*, in which, when referring to the 8^th^ case, is recorded the opinion that inflammation causes long term lesions and a cluster of symptoms. Meanwhile, it is noted that rheumatic heart may remain asymptomatic.^13^ Who was really was the very first between Chomel and Abercrombie is uncertain. However, the fact that all great physicians of the time mentioned the rheumatic heart since the 1930s, testifies the certainty that this new concept was not only an applicable idea, but a true entity in internal pathology. Elaterium was proposed as a therapeutic agent by a James Turnbull physician.^14^ Allan Webb in his 1848 work *Pathologia Indica, or The anatomy of Indian diseases* makes a review of 43 cases of rheumatic heart. All physicians recorded pericarditis and endocarditis as reasons for cardiac inflammation to cause a rheumatic heart.^15^

## EPILOGUE

Chomel was surely a pioneer in rheumatism and the first to sort the opinions of his era on rheumatic heart. He still remains among the many who had not been recognised in full for their significant contributions, desiring for a place in the history of medicine. Soon after his publication, rheumatic heart as an entity entered all internal pathology textbooks.
